# Morphological Diversity of *Gracilaria blodgettii* Harvey 1853 (Gracilariaceae, Rhodophyta) from Sarawak, Malaysian Borneo

**DOI:** 10.1155/2019/3430968

**Published:** 2019-07-02

**Authors:** Ruhana Hassan, Muhammad Nur Arif Othman, Mohd Nasarudin Harith, Amir Shah Ruddin Md Sah

**Affiliations:** ^1^Faculty of Resource Science and Technology, Universiti Malaysia Sarawak, 94300 Kota Samarahan, Sarawak, Malaysia; ^2^Centre for Pre University Studies, Universiti Malaysia Sarawak, 94300 Kota Samarahan, Sarawak, Malaysia; ^3^School of Biological Sciences, Universiti Sains Malaysia, Gelugor, 11800 Penang, Malaysia

## Abstract

*Gracilaria* red algae are notable for their economic importance as agrophytes, sold as salad vegetable, and used as the base for selected food and nonalcoholic beverages. A wild population of *Gracilaria* exists in coastal areas of Sarawak, Malaysian Borneo, but there is only limited knowledge on species diversity and its abundance leaving the untapped economic potential of this resource. This study was carried out to determine diversity of wild *Gracilaria* populations in Lawas, Santubong, and Asajaya, Sarawak, using the combination of morphological character examination and 5′ region of the mitochondrial cytochrome c oxidase 1 (CO1-5P) gene analysis. Identification of the species using morphological characters revealed three species, namely, *Gracilaria changii*, *G. blodgettii*, and *G. arcuata*, had been collected from the sampling sites. However, based on 672 bp CO1-5P gene sequence analysis, all the three species were identified as *G. blodgettii*; besides, low genetic divergence values (0.17%–0.34%) were scored between samples in this study with the same species in GenBank. In the phylogenetic trees, all samples in this study group together with other *G. blodgettii* have high bootstrap values; thus, this species is monophyletic. This study implies that species identification of *Gracilaria* and other seagrass taxa which have a phenotypic plasticity problem should include the CO1-5P gene analysis as it is a reliable gene marker for species diversity assessment.

## 1. Introduction

Genus *Gracilaria* Greville consists of more than 170 species worldwide, distributed from tropical to temperate waters, covering from intertidal to subtidal zones [1–3]. It can be found from Arctic Ocean to tropical seas of the northern hemisphere and countries of Southeast Asian regions such as Malaysia, Indonesia, Thailand, Vietnam, Singapore, and Philippines [4, 5]. *Gracilaria* is important in production of agar in food industry [6] and culture medium in research industry [7], and it serves as a habitat for various aquatic organisms [8, 9], besides becoming food for the local people.

Up to now, 20 species of *Gracilaria* had been identified in Malaysia [10], where half of them were found in Sarawak, namely, *G. arcuata* Zanardini, *G. articulata* Chang & Xia, *G. changii* Xia & Abbott, *G. coronopifolia* J. Agardh, *G. blodgettii* Harvey, *G. Salicornia* (Agardh) Dawson, *G. edulis* (Gmelin) Silva, and *G. textorii* (Suringar) Hariot and the remaining identified as *Gracilaria* sp. 1 and *Gracilaria* sp. 2 [11]. They could be found in Kuching, Bintulu, and Miri, growing in the intertidal area or attached to the man-made structures [12]. In another report, in Asajaya, Sarawak, *Gracilaria* thalli were found attached on the roots of mangrove trees [13].

Molecular studies have shown positive results in solving the identification and taxonomy of seaweeds worldwide. *Gracilaria* is known as seaweeds with high plasticity characteristics and simple morphologies with very minor variations among them and sometimes have different structures throughout its life cycles [14, 15]. Various gene markers had been used by researchers in identification of *Gracilaria* such as 5′ region of mitochondrial cytochrome c oxidase 1 (CO1-5P) [16], plastid-encoded large subunit of the ribulose-1,5-biphosphate carboxylase (*rbc*L) [17], nuclear internal transcribed spacer (ITS) [18], and intergenic spacer between the cytochrome oxidase subunits 2 and 3 (*cox*2-3 spacer) [19]. Recently, CO1-5P gene marker has been used widely due to its ability to identify red seaweed at the species level, revealing cryptic species [15, 20].

Ho et al. [21] had sequenced the nuclear and chloroplast genomes of *Gracilaria changii*. They reported that the partial nuclear genome is 35.8 Mb with 10,912 predicted proteins, while the chloroplast genome is 183,855 bp with 201 ORFs, 29 tRNAs, and 3 rRNAs. Esa [22] had produced preliminary CO1-5P gene sequences for *Caulerpa* spp. inhabiting Sarawak, and Song et al. [10] reported on microsatellite markers from expressed sequence tags (ESTs) of seaweed usage in differentiating various *Gracilaria* species, some of which had been obtained from Sarawak. Since phenotypic plasticity occurs in seaweeds, identification of *Gracilaria* species becomes a very challenging task. In this study, identification of *Gracilaria* species collected from Lawas, Santubong, and Asajaya, Sarawak, had used two approaches: (i) CO1-5P gene marker and (ii) conventional approach of identification using morphological characters.

## 2. Materials and Methods

**Figure 1:**
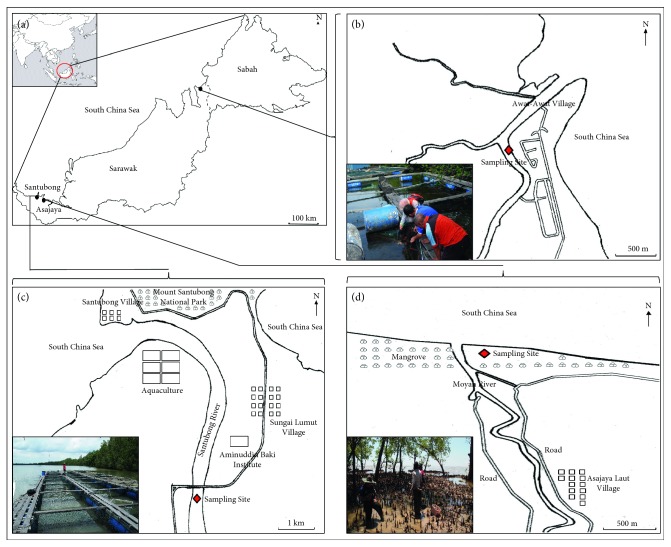
Map of (a) Sarawak, (b) Lawas, (c) Santubong, and (d) Asajaya. The diamond shape point indicates the specific location of *Gracilaria* sampling sites.

Thirteen thalli of *Gracilaria* were collected from cage culture in Santubong (01°40′42.1″N, 110°20′2.4″E) and Lawas (04°56′9.7″N, 115°14′6.8″E), while ten thalli were collected from the mangrove area in Asajaya (01°35′57.8″N, 110°36′15.9″E) (Figure 1). Identification of specimens followed keys by Dhargalkar and Kavlekar [23], Ismail [24], Lin [25], Nurridan [12], Nurridan [11], Ohmi [26], and Yamamoto [27]. The specimens were identified immediately on-site as follows: *G. changii* (GSA01-GSA10, obtained from Santubong), *G. blodgettii* (GA01-GA10, from Asajaya), and *G. arcuata* (GL01-GL03, from Lawas). Furthermore, species identification was carried out accordingly in the laboratory. During the transportation from study sites to the laboratory in the Faculty of Resource Science and Technology, Universiti Malaysia Sarawak (UNIMAS), all specimens were kept cool in the cooler box filled with ice tubes. In the laboratory, if the work cannot be done immediately, all specimens were kept in the −20°C freezer.

For molecular analysis, the samples were cleaned using distilled water to remove the epiphytes, aquatic organisms, and sand particles which may trap within the thalli. Then, each thallus was kept separately in a labelled plastic bag and stored in the −20°C freezer until further analysis. The DNA extraction of *Gracilaria* samples was done following the standard cetyl-trimethyl ammonium bromide (CTAB) protocol by Doyle and Doyle [28], followed by 1% agarose gel electrophoresis (AGE).

The amplification of CO1-5P gene was done using the polymerase chain reaction (PCR) technique with primers designed by Saunders [16]: forward primer GazF1 (5′-TCAACAAATCATAAAGATATTGG-3′) and reverse primer GazR1 (5′-ACTTCTGGATGTCCAAAAAAYCA-3′). The total volume of PCR reaction was 25 *μ*l, comprising 10.5 *μ*l ultrapure water, 4 *μ*l 10X buffer, 4 *μ*l MgCl_2_, 2.5 *μ*l dNTPs (Promega), 1.0 *μ*l forward primer GazF1, 1.0 *μ*l reverse primer GazR1, 0.5 *μ*l Taq DNA polymerase (Promega), and 1.5 *μ*l DNA template. The PCR was carried out using a PCR thermocycler (Biometra TAdvanced) under following conditions: predenaturation at 94°C for 1 minute, followed by 35 cycles of denaturation at 94°C for 1 minute, annealing at 51.3°C for 1 minute 30 seconds, extension at 72°C for 1 minute, and final extension at 72°C for 5 minutes. The success of PCR was checked using 1% AGE, and successful PCR products were sent to First Base Sdn Bhd, Selangor, Malaysia, for single-pass DNA sequencing.

CHROMAS software was used to display the CO1-5P sequences, whereas the validation of species used the basic local alignment search tool (BLAST). CLUSTAL X program (version 1.81) was used to align the DNA sequences. Neighbour joining (NJ) trees were constructed using MEGA 6.0 [31], while a model of K81uf + I + G was used for the Bayesian inference implemented on MrBayes program. All phylogenetic trees were constructed together with other Gracilariaceae species obtained from GenBank where *Janczewskia hawaiiana* and *Osmundea pinnatifida* act as outgroups (Table 1). The genetic divergence values were obtained using Kimura's two-parameter model [32].

## 3. Results

**Figure 2:**
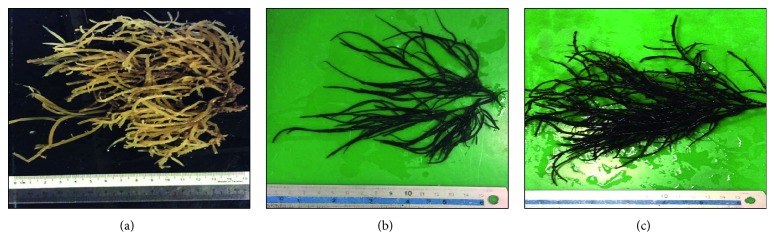
(a) Whole thallus of *G. arcuata* found in Lawas (GL01-GL03), (b) whole thallus of *G. blodgettii* found in Asajaya (GA01-GA10), and (c) whole thallus of *G. changii* found in Santubong (GSA01-GSA10).

**Figure 3:**
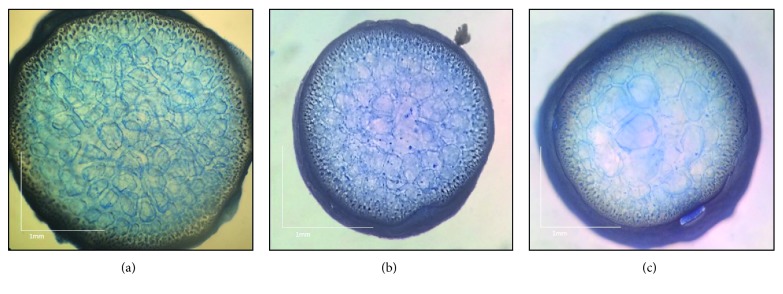
Cross section showing the medulla and cortex structure of (a) *G. arcuata*, (b) *G. blodgettii*, and (c) *G. changii*.

*Gracilaria arcuata* was found attached to the net of cage culture in Lawas. It had reddish brown colour when fresh with discoid holdfast; the branches were cylindrical, irregular, and arcuate and could grow up to 120 mm tall (Figure 2(a)). Constriction was observed at the base of every branching and the tip either pointed or divided to two to five short stubby spinose branchlets. The medulla was composed of 4-5 layers of parenchymatous cells surrounded by 2-3 layers of small cortical cells (Figure 3(a)).

*Gracilaria blodgettii* was found attached to the root of mangrove in Asajaya. The thallus was dark red in colour with discoid holdfast and could grow up to 200 mm tall (Figure 2(b)). The branching occurs frequently, either secund or irregular with constriction at the base of each branch, and it had pointed tip at the end of branches. The medulla was composed of 3-4 layers of parenchymatous cells surrounded by 2-3 layers of small cortical cells (Figure 3(b)).

*Gracilaria changii* was found attached to the net of cage culture in Santubong. The colour of *G. changii* was dark red with discoid holdfast and could grow up to 180 mm–220 mm (Figure 2(c)). The branching occurs occasionally, irregular with constriction at the base of branches. The tip either pointed or divided into two short branchlets. The medulla was composed of 3-4 layers of parenchymatous cells surrounded by 2-3 layers of small cortical cells (Figure 3(c)).

A total of 23 CO1-5P sequences had been successfully amplified with a length between 660 bp and 672 bp. Based on the BLAST results, *G. blodgettii* (GA01-GA10), *G. arcuata* (GL01-GL03), and *G. changii* (GSA01-GSA10) showed approximately 99% similarity with *G. blodgettii* mitochondrial DNA voucher with accession nos. JQ407591-JQ407596, KX017514, KX017516, KT779907-KT779908, KT779910, KT779926, and KT779928-KT779929 (Table 2). All sequences in this study had significant match with the database as each of the expect value (*E* value) was zero.

Based on genetic divergence analysis (Table 3), the three *Gracilaria* species in this study had genetic divergence values between 0% and 0.15%, when compared among each other. The genetic divergence values between the three *Gracilaria* species and the outgroups ranged from 22.60% to 24.03%. *G. blodgettii* (GA01-GA10), *G. arcuata* (GL01-GL03), and *G. changii* (GSA01-GSA10) had genetic similarity with *G. blodgettii* from China and the Philippines as mentioned in Table 2 with a variation of 0.17% to 0.35%. For comparison, high genetic variation was observed between *G. arcuata* in this study and *G. arcuata* from Japan and the Philippines within the range of 13.59%–14.90%. The comparison of intraspecific values of *G. changii* found in Santubong and Lawas with those of similar species from other countries could not be obtained due to unavailability of CO1-5P information in GenBank up to December 2018.

**Figure 4:**
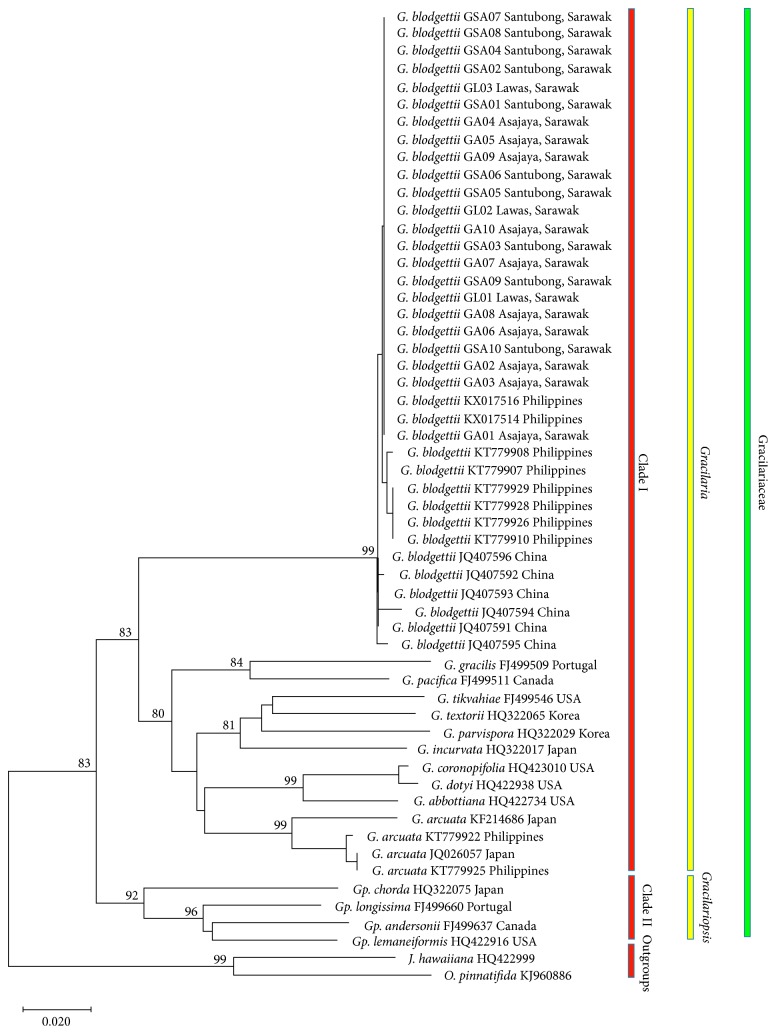
Bootstrap (50% majority rule) consensus neighbour joining tree of *G. blodgettii*, *G. arcuata*, and *G. changii* from Asajaya, Santubong, and Lawas, Sarawak, with species of Gracilariaceae acquired from GenBank and *J. hawaiiana* and *O. pinnatifida* as the outgroups. The bootstrap value of neighbour joining is indicated above the branch.

Phylogenetic trees of *Gracilaria* found in Sarawak with CO1-5P Gracilariaceae sequences from GenBank with respect to the outgroups *J. hawaiiana* and *O. pinnatifida* were successfully constructed using the Bayesian inference (not shown) and neighbour joining (Figure 4). Both trees showed similar topology, with two main clades, namely, Clade I and Clade II. Within Clade I, there are two subclades. The first subclade comprised *G. blodgettii* in this study with those from GenBank with the significant bootstrap value of 99% neighbour joining (NJ) and 1.00 Bayesian posterior probability (BPP). Therefore, *G. blodgettii* is monophyletic. The second subclade comprised all other *Gracilaria* CO1-5P gene sequences from GenBank (*G. Gracilis*, *G. pacifica*, *G*. *abbottiana*, *G. coronopifolia*, *G. dotyi*, *G. textorii*, *G. parvispora*, *G. incurvata*, *G. tikvahiae*, *G. arcuata*, and *G. changii*) with the bootstrap value of 80% (NJ) and 0.9 (BPP). Clade II consists of *Gracilariopsis* species (*Gp. chorda*, *Gp. longissimi*, *Gp. andersonii*, and *Gp. lemaneiformis*) with the strong bootstrap value of 92% (NJ) and 0.91 (BPP).

Morphological characteristic data and molecular data showed incongruent results in this study (Table 4). Three species of *Gracilaria*, namely, *G. changii*, *G. blodgettii*, and *G. arcuata*, were observed in Lawas, Santubong, and Asajaya, Sarawak, using the morphological approach, whereas only one species, namely, *G. blodgettii*, was found using the molecular approach. Summary of morphological and molecular data on *Gracilaria* obtained in this study is shown in Table 5.

## 4. Discussion

Based on morphological descriptions, samples GSA01-GSA10, obtained from Santubong, were *G. changii*; samples GA01-GA10, from Asajaya, were *G. blodgettii*; and samples GL01-GL03, from Lawas, were *G. arcuata* as they matched descriptions by Dhargalkar and Kavlekar [23], Ismail [24], Lin [25], Nurridan [12], Nurridan [11], Ohmi [26], and Yamamoto [27]. The presence of these three species in Sarawak is noted in the checklist of seaweed by Nurridan [12] and in her updated checklist in 2013. In addition, Othman et al. [9] had also reported the presence of *G. arcuata* in Lawas, Sarawak. In another study, based on morphological characters' examinations alone, Othman et al. [13] claimed that there were three species, namely, *G. changii, G. blodgettii*, and *G. coronopifolia*, found attached to the nest of cage culture in Santubong and the root of mangrove trees in Asajaya, Sarawak. Previous researchers, for example, Phang et al. [14] and Saunders [15], reported that *Gracilaria* possesses high plasticity characteristics and simple morphologies with minor variations among species and may have different structures throughout its life cycle. Thus, the morphological data of *Gracilaria* in this study should be critically examined.

In this study, CO1-5P sequences obtained were between 660 bp and 672 bp in length. Similarly, Kim et al. [20] reported that the Korean Gracilariaceae sequences had a length of 670 bp to 685 bp, while Gracilariaceae in Qingdao, China, had a length of 664 bp for CO1-5P genes [33]. All 23 sequences obtained in this study matched *G. blodgettii* from China and the Philippines with the expected value (*E* value) equal to zero. *E* value was used to determine the level of significance between DNA sequences obtained with the mitochondrial DNA voucher deposited in GenBank, where the closer the *E* value to zero, the higher the similarity of the match [34]. Therefore, all sequences in this study had significant match with the database, confirming all samples were *G. blodgettii*.

According to Kim et al. [20], Le Gall and Saunders [35], and Saunders [15], the intraspecific divergence value more than 2.0% is considered as different species. The low value of genetic divergence (0.17% to 0.35%) among these three species in Sarawak with relevant vouchers from GenBank suggested that they belong to one species, namely, *G. blodgettii*. In the phylogenetic trees, it is noted that *G. blodgettii*, *G. arcuata*, and *G. changii* in this study with *G. blodgettii* from GenBank (Clade I, the first subclade) had recorded a high bootstrap support with values of 99% (NJ) and 1.00 (BPP). The strong value of bootstrap support given to this clade means it is very likely that the relationship is true, further supporting that all specimens belonged to the same species.

According to Saunders [15], misidentification of *Gracilaria* species may likely to happen due to its simple and high plasticity characters. Zhao et al. [33] reported that not all red seaweeds could be identified based on the morphological approach, especially Gracilariaceae due to the following reasons: (i) they have high varieties of morphologies within the species; (ii) they have simple morphological characteristics and highly convergent morphology which make them look similar to others leading to confusion during identification; (iii) lack of distinct parts or features to differentiate among species; and (iv) their reproduction cycle is very complex due to heteromorphic alternation of generation. Similarly, Md Sah et al. [14] also claimed that the identification of *Gracilaria* could be problematic because of limitations of distinct morphological and reproductive characteristics. For example, in Virginia, Thomsen *et al.* [36] had corrected the identification of *G. vermiculophylla* which at first has been referred to as *G. verrucosa* and *G. tikvahiae*. Meanwhile, *G. vermiculophylla* in British Columbia was overlooked because of similar characteristics of *Gracilaria* species that exist there [15]. There are also cases where the same species have different characteristics because of environmental factors that lead to confusion during identification [37, 38].

In this study, identification of *Gracilaria* species (*G. blodgettii*, *G. arcuata*, and *G. changii*) using the morphological approach most likely resulted in misidentification because of either a variety of morphologies, complexity of the life cycle, or the environments where they grow. In addition, to avoid further confusion, Thomsen et al. [36] suggested that each specimen should be kept properly using air-drying or herbarium-pressing methods so that future studies could refer to them as reference materials. Guiry and Guiry [3] also reported in their taxonomic note that the samples identified as *G. blodgettii* should be compared with the topotype material from Key West, Florida, USA, for confirmation and avoiding further misidentification. For now, the sequences of *Gracilaria* samples in Sarawak, Malaysian Borneo, should be identified as *Gracilaria aff. blodgettii* until we could compare the samples with *G. blodgettii* from Key West, Florida, USA. Thus, the study on seaweed identification should be continued to resolve the taxonomy and create clear understanding on their morphology including plasticity characteristics that respond to environmental changes.

## 5. Conclusion

This study suggests that three *Gracilaria* species obtained in Lawas, Santubong, and Asajaya, Sarawak, which were initially identified as *G. blodgettii*, *G. arcuata*, and *G. changii*, should be renamed into one species, namely, *Gracilaria blodgettii* Harvey 1853. Minor differences between specimens in terms of morphology may be due to environmental influence. For future seaweed research, CO1-5P gene markers should be sequenced and analysed, besides the conventional methods of using species identification keys provided by previous researchers.

## Figures and Tables

**Table 1:** List of Gracilariaceae and other species analysed in this study.

Species	Locality	Collection date	No. of samples	Field voucher	GenBank
*G. changii*	Santubong, Sarawak	October 2014	10	GSA01-GSA10	—
*G. blodgettii*	Asajaya, Sarawak	December 2014	10	GA01-GA10	—
*G. arcuata*	Lawas, Sarawak	November 2014	3	GL01-GL03	—
*G. abbottiana* M. D. Hoyle	Hawaii, USA [29]	—	—	—	HQ422734
*G. coronopifolia* J. Agardh	Hawaii, USA [29]	—	—	—	HQ423010
*G. dotyi* Hoyle	Hawaii, USA [29]	—	—	—	HQ422938
*G. gracilis* (Stackhouse) Steentoft, L. M. Irvine, & Farnham	Portugal [15]	—	—	—	FJ499509
*G. incurvata* Okamura	Misaki, Japan [20]	—	—	—	HQ322017
*G. pacifica* I. A. Abbott	British Columbia, Canada [15]	—	—	—	FJ499511
*G. parvispora* I. A. Abbott	Jeju, Korea [20]	—	—	—	HQ322029
*G. textorii* (Suringar) Hariot	Jeju, Korea [20]	—	—	—	HQ322065
*G. tikvahiae* McLachlan	Rhode Island, USA [15]	—	—	—	FJ499546
*G. blodgettii* Harvey	Philippines (unpublished data)	—	—	—	KX017514-KX017516, KT779907-KT779908, KT779910, KT779926, KT779928, KT779929
*G. blodgettii* Harvey	China (unpublished data)	—	—	—	JQ407591-JQ407596
*G. arcuata* Zanardini	Japan (unpublished data)	—	—	—	JQ026057
*G. arcuata* Zanardini	Japan [30]	—	—	—	KF214686
*G. arcuata* Zanardini	Philippines (unpublished data)	—	—	—	KT779922
*G. arcuata* Zanardini	Philippines (unpublished data)	—	—	—	KT779925
*Gp. andersonii* (Grunow) E. Y. Dawson	British Columbia, Canada [15]	—	—	—	FJ499637
*Gp. chorda* (Holmes) Ohmi	Misaki, Japan [20]	—	—	—	HQ322075
*Gp. lemaneiformis* (Bory de Saint-Vincent) E. Y. Dawson, Acleto, & Foldvik	Hawaii, USA [29]	—	—	—	HQ422916
*Gp. longissima* (S. G. Gmelin) Steentoft, L. M. Irvine, & Farnham	Ria de Aveiro, Portugal [15]	—	—	—	FJ499660
*J. hawaiiana* Apt	Hawaii, USA [29]	—	—	—	HQ422999
*O. pinnatifida* (Hudson) Stackhouse	Brittany, France (unpublished data)	—	—	—	KJ960886

**Table 2:** Summary of BLAST results for all CO1-5P sequences obtained in this study.

Species name	Voucher no.	Similarity toward other organism	Accession no.	Maximum identical (%)	*E* value
*G. arcuata*, *G. blodgettii*, *G. changii*	GL01-GL03, GA01-GA10, GSA01-GSA10	*G. blodgettii*	JQ407591-JQ407596, KX017514, KX017516, KT779907-KT779908, KT779910, KT779926, KT779928-KT779929	99	0.0

**Table 3:** Genetic distance (%) based on CO1-5P gene sequence analysis in this study.

	Species	1	2	3	4	5
1	*G. blodgettii*	—				
2	*G. arcuata*	0.15	—			
3	*G. changii*	0.15	0.15	—		
4	*J. hawaiiana*	22.60	22.60	22.60	—	
5	*O. pinnatifida*	24.03	24.03	24.03	10.67	—

**Table 4:** Comparison of *Gracilaria* species based on morphological characteristics and CO1-5P gene markers.

Locations	Name of the species	
	Morphological characteristics	Molecular CO1-5P gene analysis
Santubong, Sarawak	*G. changii*	*G. blodgettii*
Lawas, Sarawak	*G. arcuata*	*G. blodgettii*
Asajaya, Sarawak	*G. blodgettii*	*G. blodgettii*

**Table 5:** Summary of morphological and molecular data on *Gracilaria* obtained in this study.

Species	Location	Morphological characteristics	Similarity toward data in GenBank (%)	Intraspecific divergence from similar locations (%)	Genetic divergence with *G. blodgettii* in GenBank using CO1-5P (%)	Genetic divergence with similar species in GenBank using CO1-5P (%)
*G. arcuata*	Cage net, Lawas	Reddish brown, discoid holdfast, cylindrical, irregular, and arcuate branching, tip pointed or 2-5 short branchlets, constricted at base, 4-5 layers of medulla, 2-3 layers of cortex	99 (*G. blodgettii*)	0.15	0.17–0.35	13.59–14.90
*G. changii*	Cage net, Santubong	Dark red, discoid holdfast, branching occasionally and irregularly, constricted at base, tip pointed or two short branchlets, 3-4 layers of medulla, 2-3 layers of cortex	99 (*G. blodgettii*)	0.15	0.17–0.35	Not available
*G. blodgettii*	Mangrove, Asajaya	Dark red, discoid holdfast, branching frequently, secund or irregular, constricted at base, pointed tip, 3-4 layers of medulla, 2-3 layers of cortex	99 (*G. blodgettii*)	0.15	0.17–0.35	0.17–0.35

## Data Availability

The data used to support the findings of this study are available from the corresponding author upon request.
